# Total ankle prostheses in rheumatoid arthropathy: Outcome in 52 patients followed for 1–9 years

**DOI:** 10.3109/17453670903153568

**Published:** 2009-08-01

**Authors:** Huub JL van der Heide, Bernard Schutte, Jan Willem K Louwerens, Frank H J van den Hoogen, Maarten C de Waal Malefijt

**Affiliations:** ^1^Department of Orthopaedics, Radboud University, Nijmegen Medical CentreNijmegenthe Netherlands; ^2^Department of Orthopaedics, Nijmegenthe Netherlands; ^3^Department of Rheumatology, MaartenskliniekNijmegenthe Netherlands

## Abstract

**Background and purpose** The first generations of total ankle replacements (TARs) showed a high rate of early failure. In the last decades, much progress has been made in the development of TARs, with the newer generation showing better results. We evaluated TARs implanted with rheumatoid arthritis (RA) or juvenile inflammatory arthritis (JIA) as indication.

**Patients and methods** 58 total ankle prostheses (Buechel-Pappas and STAR type) were implanted in patients with RA (n = 53) or JIA (n = 5) in 54 patients (4 bilateral). After a mean follow-up of 2.7 (1–9) years, all patients were reviewed by two orthopedic surgeons who were not the surgeons who performed the operation. Standard AP and lateral radiographs were taken and a Kofoed ankle score was obtained; this is a clinical score ranging from 0–100 and consists of sub-scores for pain, disability, and range of motion.

**Results** 2 patients died of unrelated causes. Of the 52 patients who were alive (56 prostheses), 51 implants were still in place and showed no signs of loosening on the most recent radiographs. The mean Kofoed score at follow-up was 73 points (SD 16, range 21–92). 4 patients showed a poor result (score < 50) with persistent pain for which no obvious reason could be found. 5 implants were removed, 4 because of infection and 1 because of aseptic loosening.

**Interpretation** Medium-term results of the STAR and BP types of TAR in RA were satisfactory. The main reason for failure of the implant was infection.

## Introduction

The standard treatment for end-stage arthritis of the ankle joint due to rheumatoid arthritis (RA) has been an ankle fusion. In the last decades, much progress has been made in the development of total ankle replacements (TARs). Most TARs are implanted in patients with RA and posttraumatic arthritis. The first generations of TARs were implants with 2 parts, one inserted in the distal tibia and one placed on top of the talus. These implants showed a high rate of early failure, especially early loosening. The current TARs consist of three components, a metal implant placed in the distal tibia, a metal component in the talus, and a polyethylene insert in-between. These implants allow a certain amount of gliding and rotation, whereas the previous more constrained prostheses behaved as a hinge.

This study was undertaken to evaluate all TARs implanted at our centers in patients with RA or juvenile inflammatory arthritis (JIA) as indication.

## Patients and methods

### Patient characteristics (Figure 1)

Between 1996 and 2004, 58 total ankle prostheses were implanted in 54 patients with RA or JIA (53 and 5 TARs, respectively; 4 bilateral) at the Radboud University Nijmegen Medical Center and Sint Maartenskliniek, both in Nijmegen, the Netherlands. 48 implants were placed in women, and 10 in men. Mean age at the time of operation was 55 (27–82) years. All patients were operated by experienced senior orthopedic surgeons (JWL, MdWM) using the same technique.

**Figure 1.**
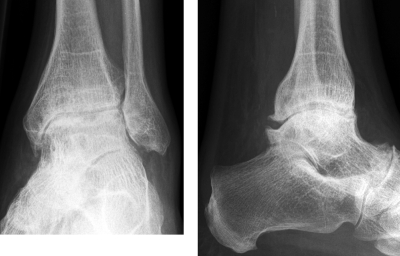
Severe degenerative changes.

### Types of implants and surgical technique

**Figure 2.**
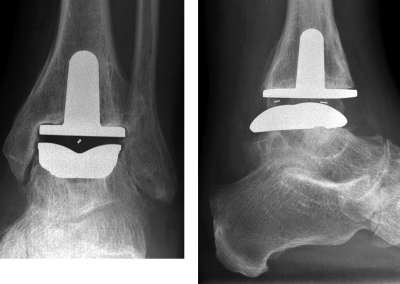
A Buechel-Pappas type ankle prosthesis in situ.

Two different implants were used, both consisting of 2 metal components and a polyethylene bearing. One surgeon (JWL) always used the Scandinavian-Type Ankle Replacement (STAR, Waldemar Link, Hamburg, Germany) (n = 29), while the other surgeon (MdWM) used the same implant for 3 years (n = 8); the rest of the implants were Buechel-Pappas type (BP; Endotec, South Orange, NJ) (n = 21) (Figure 2).

After antibiotic prophylaxis (1 gram Cefazolin i.v.) and placement of a tourniquet, an anterior approach was used. After mobilization of the neurovascular bundle to the lateral side, the joint was opened and the synovium excised. The bony surfaces of the distal tibia and the talus were prepared for placement of the 2 metal components, whereafter the polyethylene bearing was inserted in-between. The wound was closed over a suction drain, which was removed on the second postoperative day. After the postoperative swelling had diminished, a lower leg cast was applied. Antithrombotic prophylaxis was by means of 0.3 mL nadroparin subcutaneously for 6 weeks. Patients started weight bearing after 2 weeks, when a walking cast was applied. After 4 weeks, the cast was removed and patients could start to ambulate as tolerated and to practice movement of the artificial joint. Normally, physiotherapy was not needed.

### Outcome measures

After a mean follow-up of 2.7 (1–9) years, all patients were reviewed by 2 orthopedic surgeons who were not the surgeons who performed the operation. Standard AP and lateral radiographs were taken and a Kofoed score was obtained. We analyzed 2 forms of failure: firstly, the traditional failure rate consisting of removal of the prosthesis or amputation. The second endpoint was defined as removal of the prosthesis for any reason or a clinical score below 50.

## Results

### Complications

During implantation of the prostheses, 10 cases sustained a medial malleolar fracture, 3 a lateral malleolar fracture, and in 3 patients a fracture of the posterior part of the tibial plafond occurred. The occurrence of a fracture was unrelated to the type of implant used, the surgeon, or the total number of implants the surgeon had placed before. The posterior tibial plafond fractures and 6 medial malleolar fractures were treated with the normal aftercare (6 weeks with below-knee walking cast) and healed uneventfully. The 3 lateral malleolar fractures and the remaining medial fractures were treated with reduction and internal fixation (malleolar screw on the medial side; lag screw and neutralization plate on the lateral side).

One implant became infected after a wound-healing problem over a medial malleolar fracture, which had been treated with osteosynthesis. This implant was removed and the ankle was fused 6 months after the index operation. Another implant lost its tibial fixation and had to be removed; a fusion was performed subsequently. The remaining 14 fractures healed uneventfully. 3 patients had early surgical site infections that were treated by exploration of the surgical site and debridement of the wound, and systemic and local antibiotics. 2 early infections were treated successfully. In the other patient, the implant had to be removed and an ankle fusion was performed.

Two chronic infections occurred, the first was the above-mentioned patient with a wound-healing problem over a fracture. Initially, the wound appeared to heal well but after a few weeks the patient developed a deep infection, which was treated by removal of the implant and fusion of the ankle, 6 months after the index operation. Another late infection was seen 4 years after implantation of the TAR; this patient had an ipsilateral infected total knee replacement. Both the infected knee prosthesis and the TAR could not be treated adequately with antibiotics and several surgical debridements, and she underwent an above-knee amputation.

One STAR talar component showed symptomatic migration and was removed after 4.2 years, and the ankle was subsequently fused.

A summary of all complications is given in Table 1.

**Table 1.** Summary of complications and outcome.

Complication **^a^**	Resulted in failure (5)	Implant in situ (51)
Peroperative fracture	2	14
Early infection	1	2
Late infection	2	0
No perioperative complications	1	35

**^a^** One patient had both an infection and a fracture.

### Outcome

2 patients died of an unrelated cause 4 and 4.3 years after unilateral TAR operations; at the last follow-up there had been no radiographic signs of loosening.

In the 52 patients who were alive at the latest follow-up (56 prostheses), 51 implants were still in place after a mean follow-up of 2.7 (1–9) years. The mean Kofoed ankle score at follow-up was 73 points (SD 16, range 21–92). 4 patients showed a poor result (with a Kofoed score of < 50). These 4 patients all had persistent pain, for which no obvious reason could be found (Table 2).

**Table 2.** Results in relation to the type of implant used.

	STAR (n = 37)	BP (n = 21)
Poor clinical result		
(Kofoed score < 50)	4	0
Removal of implant	4	1
Implant in situ and Kofoed > 50	29	20
Mean (range) follow-up in years	2.4 (0.5–6.5)	3.3 (0.5–9)
Died from unrelated cause	2	0

**Figure 3.**
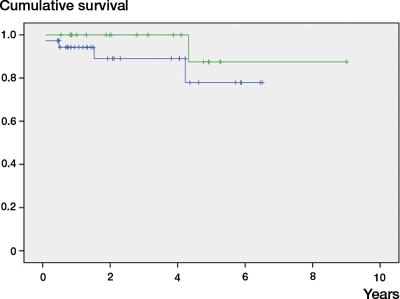
Kaplan-Meier survival curve with removal of the prosthesis as outcome measure. The green line represents the BP prosthesis, the blue line the STAR.

With removal of the implant as endpoint, the success rate was over 92% (Figure 3). Taking clinical failure (Kofoed score < 50) into account, the success rate was 88%. A logistic regression analysis with implant failure as dependent variable showed that infection was the most important factor (p < 0.001). Type of implant (p = 0.08), surgeon (p = 0.4), peroperative fracture (p = 0.6), side operated (p = 0.7), and sex (p = 0.3) did not reach statistical significance. Logistic regression analysis with implant failure or a clinical score below 50 as endpoint showed the same results: infection was the only significant predictive factor (p < 0.01); type of implant (p = 0.08), surgeon (p = 0.8), peroperative fracture (p = 0.2), side operated (p = 0.6), and sex (p = 0.08) were not significant.

The relative risk (RR) of implant failure with or without infection was 15 (CI: 2–45). The RR of failure with or without a perioperative fracture was 1.66 (CI: 0.3–9).

## Discussion

Total ankle replacement in RA showed good medium-term results in our series, similar to those in a recently published meta-analysis (Stengel et al. 2005). Early and especially late infection of the prosthesis is a major threat. The high incidence of peroperative fractures did not influence the outcome. It is important to recognize that complications such as peroperative fracture do not necessarily lead to a poor outcome; on the other hand, the absence of complications or radiographic findings does not guarantee a good clinical result. In many studies regarding the medium- or long-term results of endoprostheses, the endpoint has been revision for any reason. Failure may also be defined as patient dissatisfaction with the result of the procedure, regardless of the symptoms or physical findings (Hasan et al. 2002).

Rheumatoid arthritis frequently involves the hindfoot (Michelson et al. 1994, Klenerman 1995, Cracchiolo 1997). Unfortunately, this part of the foot is often overlooked when caring for these patients—who often have multiple areas of involvement giving pain and producing deformity. Early treatment consists of proper or custom-made footwear and occasional injections of corticosteroids (Cracchiolo 1997). Early synovectomy may be valuable, but it is controversial whether a synovectomy can halt the progression of joint destruction (Akagi et al. 1997, Nakamura et al. 2004). Ankle fusion is a well-established operation with few complications and good pain relief, and improvement in walking ability (Maenpaa et al. 2001, Panotopoulos et al. 2005, Winson et al. 2005).

With an arthrodesis of the talotibial joint, the normal function of the hindfoot is altered. This results in altered walking kinematics—especially an earlier heel-off—and this becomes worse when walking barefoot or with increased speed (Beyaert et al. 2004). The loss of ankle motion caused by an ankle fusion increases strain on the adjacent joints of the ipsilateral foot, and many patients develop degenerative changes in the subtalar and midtarsal joints (Mazur et al. 1979, Muir et al. 2002). It is important to know that in the literature prior to 1979, the results obtained with ankle arthrodesis were generally graded as good if arthrodesis was achieved or poor if nonunion resulted, so the result “good” or “poor” was only based on the radiograph; the clinical result was not taken into account (Abidi et al. 2000).

The advantages of total ankle replacement over ankle arthrodesis include preservation of motion and reduced stresses on the midfoot and subtalar joints (Su et al. 2004). Especially patients with rheumatoid arthritis may already have subtalar degeneration when they present with ankle pain, making ankle arthrodesis a less appealing treatment. Secondary procedures to the foot, and especially the subtalar joint, are often part of the surgical plan and are performed either before or simultaneously with ankle replacement (Greisberg et al. 2004).

The early TAR design components were highly constrained; these implants showed an unacceptable rate of loosening—as high as 50% within a few years (Dini and Bassett 1980, Newton 1982, Helm and Stevens 1986). This high failure rate is probably due to the greater stress at the bone-prosthesis interface, as constrained implants do not allow rotation and sliding during extension and flexion (Conti and Wong 2001). The current generation of implants, consisting of 3 components including a mobile polyethylene bearing, shows a lower loosening rate after medium-term follow-up (Buechel and Pappas 1992, Kofoed and Sorensen 1998, Kofoed and Lundberg-Jensen 1999, Buechel et al. 2002, Anderson et al. 2003, Buechel et al. 2003, Kofoed 2004, Su et al. 2004, Doets et al. 2006, San Giovanni et al. 2006). Both implants used in our series are well documented. The STAR prosthesis has shown comparable results in other studies (Kofoed and Sorensen 1998, Kofoed and Lundberg-Jensen 1999, Anderson et al. 2003, Wood and Deakin 2003, Kofoed 2004), as has the Buechel-Pappas type (Buechel and Pappas 1992, Buechel et al. 2002, Buechel et al. 2003, Su et al. 2004, Doets et al. 2006, San Giovanni et al. 2006). Most studies regarding the results of implants have been from the institute of the designer of the implant. The experience of these individuals, while valuable, is not necessarily representative of the experience of orthopedic surgeons in general. Furthermore, in most outcome studies different indications are included; in our study only RA and JIA patients were included.

In a radiostereometric analysis of the tibial component of the Buechel-Pappas type of implant, a migration of the tibial component of 0.9 mm was observed during the first 3 months, but this stabilized by the 6-month follow-up (Nelissen et al. 2006). We are not aware of any reports regarding the initial stability of the talar component of the BP prosthesis. For the STAR type, a similar migration pattern was found: some migration during the initial postoperative period but little or no migration after 6 weeks (Carlsson et al. 2005).

Although complications are commonly described and the surgery is technically demanding (Greisberg et al. 2004), in experienced hands and with a good indication, this kind of surgery may yield good results. However, a suboptimal placement of the prosthesis will lead to early failure (Greisberg et al. 2004). The results for patients with degeneration due to osteoarthritis do not differ from those suffering from rheumatoid arthritis (Kofoed and Sorensen 1998); furthermore, age does not seem to affect the outcome (Kofoed and Lundberg-Jensen 1999).

Compared to a standard ankle fusion, ankle fusion after a failed arthroplasty more often results in a non-union (Hopgood et al. 2006, Kotnis et al. 2006). In our group, 2 patients showed a delayed union—for which a new intervention was necessary, but all fusions united. Although the function of a total ankle prosthesis is never the same as a normal ankle joint and clinical results are sometimes poor when looking at a subjective score, patient satisfaction is higher than expected from these scores (Nishikawa et al. 2004).
